# Colorimetric cadmium ion detection in aqueous solutions by newly synthesized Schiff bases

**DOI:** 10.3906/kim-1912-36

**Published:** 2020-06-01

**Authors:** Ziya AYDIN, Mustafa KELEŞ

**Affiliations:** 1 Vocational School of Technical Sciences, Karamanoğlu Mehmetbey University, Karaman Turkey; 2 Department of Chemistry, Faculty of Arts and Sciences, Osmaniye Korkut Ata University, Osmaniye Turkey

**Keywords:** Colorimetric, Schiff base, sensor, cadmium

## Abstract

Two newly synthesized Schiff bases DMCA and DMBA were used for selective detection of Cd^2+^ over a wide range of other metal ions in acetonitrile (ACN)/ Tris-HCl buffer (10 mM, pH 7.32, v/v 2:1). The sensors can detect Cd^2+^ ions by colour changes from colourless to orange for DMBA and yellow to reddish for DMCA. Response of the probes towards metal ions was investigated by using UV-vis spectroscopy. The complex stoichiometry between the sensors, DMBA and DMCA, and Cd^2+^ was found to be 2:1 and the binding constants were calculated to be 2.65 ×10^12^ M^-2^ and 4.95 ×10^12^ M^-2^, respectively. The absorbance-based detection limits of DMBA and DMCA were calculated as 0.438 μM and 0.102 μM, respectively. The sensors were also successfully applied to real samples.

## 1. Introduction

As one of the highly toxic heavy metal ions, cadmium is widely distributed in water, soil and crops, generated from its use sources such as fertilizers, the combustion of fossil fuels, paint pigments, Ni-Cd rechargeable batteries, causing serious problems for human health [1–3]. Due to the high affinity to sulphur, Cadmium ion (Cd^2+^) can interfere with Ca^2+^ and Zn^2+^ in the binding sites of some enzymes containing sulphur [4,5]. It causes these enzymes to malfunction, causing severe organ damage. Cadmium and cadmium compounds are category I carcinogens [6], and are known to be associated with liver and kidney damage, cancer mortality and cardiovascular disease [7–9]. Thus, it is an essential point to develop detection methods for cadmium. Several methods have been reported to detect Cd^2+^ ; however, these methods are generally expensive, time consuming and have sophisticated synthetic procedures [10–13]. As alternative methods, fluorometric and colorimetric sensors require easier procedures. In recent years, fluorescent sensors have gained growing interest in detecting Cd^2+^ ions [14–17]. Many of them, however, have some limitations such as having a poor detection limit [18] and complicated synthesis steps [19]. Moreover, most of the sensors for Cd^2+^ also give response to Zn^2+^ ions due to their similar properties [20]. Recently, several colorimetric sensors for Cd^2+^ have been also reported [21–24]. These sensors also suffer from long response time, poor selectivity, complex organic synthesis and poor detection limits [25]. Nowadays, Schiff base derivatives have been increasingly used as colorimetric sensors due to having simple synthesis steps and high selectivity. Especially, cinnamaldehydebased and benzaldehyde-based Schiff base derivatives have been developed for the detection of metal ions such as Ni^2+^ [26], Ag^+^ [27], Al^3+^ [28,29] Cu^2+^ /Hg^2+^[30]. However, simple, rapid, highly selective, and sensitive colorimetric sensors for Cd^2+^ are still rare and needed to be developed. In this paper, we presented 2 newly synthesized Schiff base derivatives, one of them is a benzaldehydebased sensor and the other is a cinnamaldehyde-based sensor, which detected Cd^2+^ ions by colour change in aqueous solutions. Response of the sensors was investigated using UV-vis spectroscopy in the presence of various metal ions. The detection limits of DMBA and DMCA were calculated to be 0.438 μM and 0.102 μM, respectively.

## 2. Experimental

### 2.1. Materials and methods

N-phenyl-o-phenylenediamine, 4-(dimethylamino)cinnamaldehyde and 4-(dimethylamino)benzaldehyde were purchased from Sigma-Aldrich. The solvents and the other chemicals used in the experiments were commercially obtained. The solution of Fe^3+^ and Fe^2+^was prepared separately by dissolving in 0.1 M HCl. Unless otherwise stated, the solutions of the metal ions tested were prepared from nitrate salts or chloride salts of them in deionized water. A NMR spectrometer (a Bruker NMR spectrometer (Bruker Ultrashield Plus Biospin Avance III 400 MHz NaNoBay FT-NMR)) was used to record ^1^H and ^13^C NMR spectra. An Agilent LC-MS/MS 6460Triple Quadrupole mass spectrometer was used to perform ESI-MS analyses. Shimadzu UV-1800 spectrophotometer was used to record UV-vis spectra.

### 2.2. Synthesis and characterization of the probes

**Synthesis of N,N-dimethyl-4-(((2-(phenylamino)phenyl)imino)methyl)aniline (DMBA):**

4-(Dimethyl)aminobenzaldehyde (300 mg, 2 mmol) and N-phenyl-o-phenylenediamine (369 mg, 2 mmol) were dissolved in ethanol (5 mL), respectively. The solutions were charged in Schlenk tube and mixed for 1h. Then, a pale-yellow precipitate product was filtered and washed with cold ethanol to obtain DMBA. Yield 0.510 g (81%). ^1^H NMR (400 MHz, CDCl_2_) δ 8.34 (s, 1H), 7.76 (d, J = 8.8 Hz, 2H), 7.25 (dd, J = 7.1, 6.^1^Hz, 2H), 7.21–7.14 (m, 2H), 7.07 (ddd, J = 16.2, 6.7, 5.2 Hz, 4H), 6.94–6.85 (m, 1H), 6.72 (d, J = 8.9 Hz, 2H), 5.73 (s, 1H), 3.03 (s, 6H). ^13^C NMR (100MHz, CDCl_2_) δ 158.3, 152.4, 146.5, 143.7, 140.6, 130.3, 129.4, 124.0, 120.6, 119.2, 117.3, 111.7, 111.1, 40.2. ESI-MS (positive mode) m/z 316.2 [DMBA+H]^+^.

**Synthesis of N,N-dimethyl-4-((1E,3E)-3-((2-(phenylamino)phenyl)imino)prop-1-en-1-yl)aniline (DMCA):**

4-(Dimethylamino)cinnamaldehyde (350 mg, 2 mmol) and N-phenyl-o-phenylenediamine (369 mg, 2 mmol) were dissolved in ethanol (5 mL), respectively. The solutions were charged in Schlenk tube and mixed for 1h. Then, the reddish precipitate product was filtered and washed with cold ethanol to obtain DMCA. Yield 0.580 g (85%). 1H NMR (400 MHz, CDCl_2_) δ 8.27 (d, J = 8.7 Hz, 1H), 7.49–7.33 (m, 2H), 7.33–7.23 (m, 3H), 7.21–7.11 (m, 2H), 7.12–7.01 (m, 5H), 7.01–6.87 (m, 2H), 6.68 (t, J = 12.2 Hz, 2H), 5.76 (s, 1H), 3.02 (s, 6H). ^13^C NMR (100MHz, CDCl_2_) δ 159.9, 151.3, 144.0, 143.3, 141.2, 129.4, 129.0, 124.1, 122.1, 120.9, 118.7, 117.7, 112.1, 111.8, 40.2. ESI-MS (positive mode) m/z 342.3 [DMCA+H]^+^.

### 2.3. UV-vis absorption measurements

DMBA (3.15 mg, 0.01 mmol) and DMCA (3.42 mg, 0.01 mmol) were dissolved in ACN (10 mL) and 30 μL of the sensors (1 mM) were diluted with 1.470 mL ACN/Tris-HCl buffer (10 mM, pH 7.32, v/v 2:1) to make final concentrations of 20 μM. For each spectrum, 1.0 mL of a probe solution was added to a 1-cm quartz cell, to which different stock solutions of cations were gradually added by using a micro-pipette. All absorption spectra were collected from 220 to 800 nm. Upon addition of each metal ions tested to the sensors solutions, the spectral readings were immediately recorded.

### 2.4. Determination of Cd^2+^in real samples

To evaluate the analytical applicability of the sensors, DMBA and DMCA, they were used to detect Cd^2+^ions in tap water samples collected in Osmaniye, Turkey. The tap water samples were spiked with solutions of Cd^2+^and were diluted with ACN/Tris-HCl buffer (10 mM, pH 7.32, v/v 2:1) to obtain samples at concentrations of 0, 2, 5, and 10 mg. L−1 (ppm) Cd^2+^, respectively. All spectroscopic measurements were done under the same experimental conditions proposed for the selectivity experiments, and measurements were performed at least triplicate and resulting averages were reported.

## 3. Results and discussion

### 3.1. Design and synthesis of the sensors, DMBA and DMCA

**Scheme 1:**
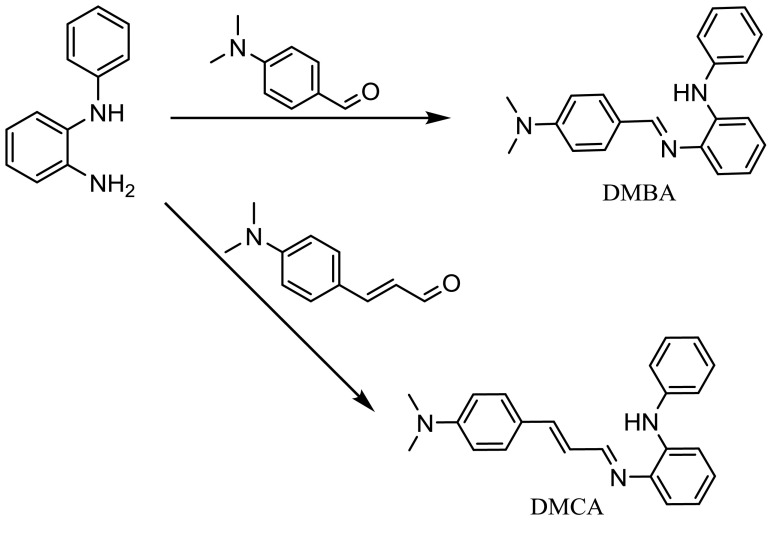
Synthesis of the probes, DMBA and DMCA.

The molecular structures of the sensors were designed to contain an N-phenyl-o-phenylenediamine as a binding part for Cd^2+^, a cinnamaldehyde moiety (for DMCA), and a benzaldehyde moiety (for DMBA) as chromophore parts. The binding parts of the sensors consist of 2 nitrogen atoms to give 2 5-membered rings in 2:1 binding between the sensors and Cd^2+^. The binding part and the chromophore parts were linked via the formation of the C=N bonds in a 1-step procedure with 81% and 86% yields for DMBA and DMCA, respectively (Scheme 1). The structures of the sensors were verified by NMR (^13^C NMR and ^1^H NMR) and ESI-mass spectrometry.

### 3.2. The absorption and colorimetric properties of DMBA and DMCA

**Figure 1:**
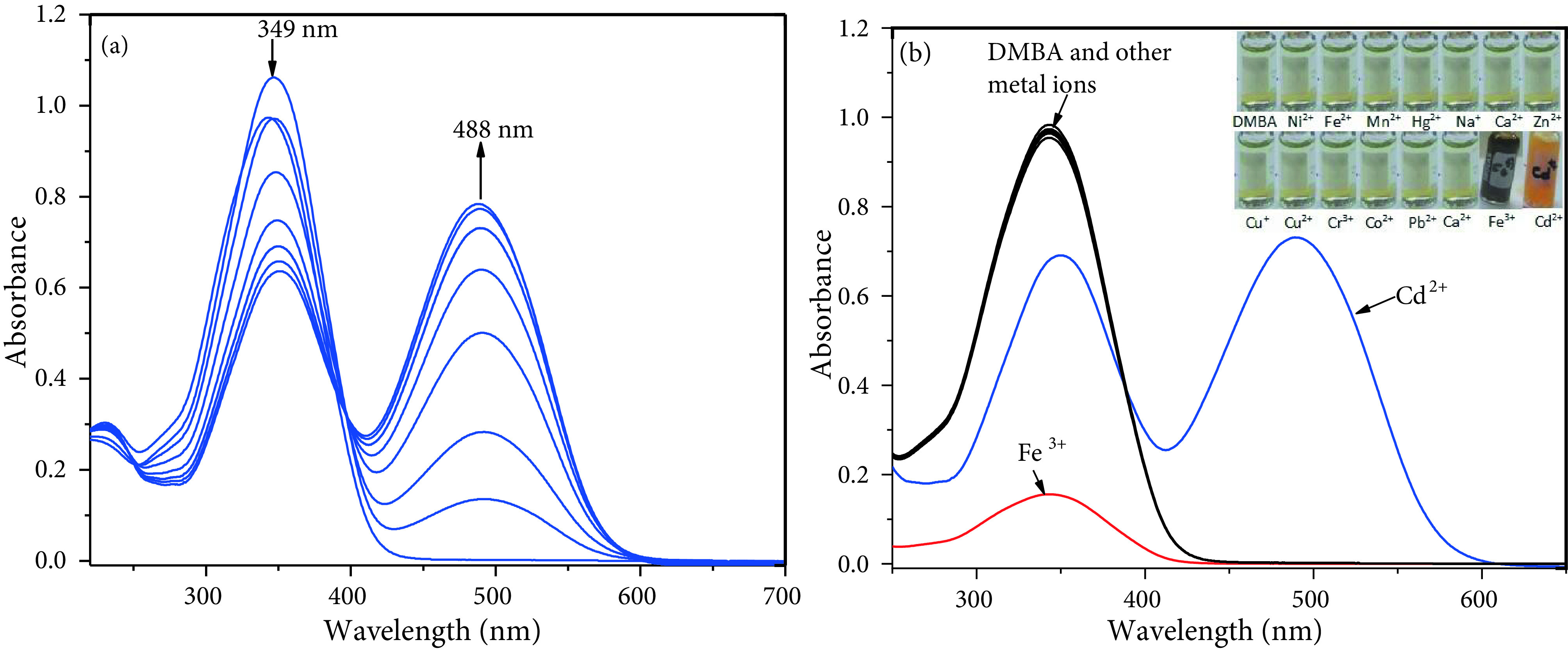
(a) Absorption spectra of 20 μM DMBA with gradual addition of CdCl2 (0, 2, 4, 6, 8, 10, 12, 14, 16, 18, 20, 25, 30, 35, and 40 μM, respectively) in ACN/Tris-HCl buffer (10 mM, pH 7.32, v/v 2:1) (b) Absorption spectra of DMBA (20 μM) with various metal ions (20 μM for Cd^2+^, Cu^2+^, Ni^2+^, Mn^2+^, Hg^2+^, Zn^2+^, Ag^+^, Pb^2+^, Fe^3+^, Co^2+^, Fe^2+^, Cu^+^ and Cr^3+^; 50 μM for Ca^2+^, Mg^2+^, K^+^, and Na^+^) in ACN/Tris-HCl buffer (10 mM, pH 7.32, v/v 2:1) (inset: colour changes of DMBA with the metal ions tested)

We first evaluated the spectroscopic properties of DMBA and DMCA and their interactions with various metal ions. The colourless compound DMBA (20 μM) in ACN/Tris-HCl buffer (10 mM, pH 7.32, v/v 2:1) displays a maximum absorption at 349 nm (ε = 4.75 ×104 M^-1^ cm^-1^, only DMBA) that may be ascribed to n-π* transition [31]. However, the addition of Cd^2+^resulted in a decrease in the absorption intensity at 349 nm and formation of a new absorption peak at 488 nm (Figure 1a) with a remarkable colour change from colourless to orange (Figure 1b inset). As depicted in Figure 1b, the tested metal ions including Cu^2+^ , Cr^3+^, Cu^+^, Na^+^, Hg^2+^, Mg^2+^, Ca^2+^, Zn^2+^ , Ag^+^, Pb^2+^, K^+^, Co^2+^, Fe^2+^, Mn^2+^ , and Ni^2+^ did not respond to DMBA while Fe^3+^ caused a decreasing the absorption intensity at 349 nm without any increase at 488 nm.

**Figure 2:**
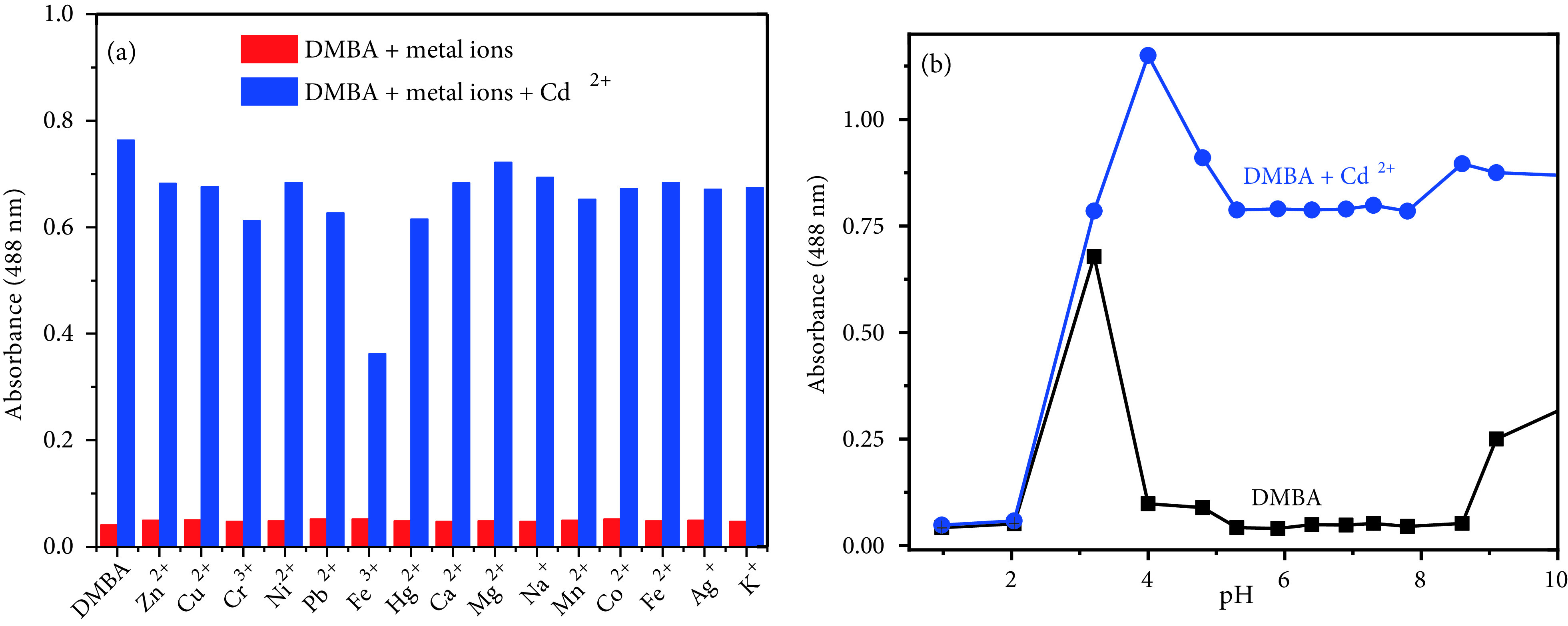
(a) Absorbance intensities of DMBA (20 μM) with various metal ions (200 μM) (red bar) and the subsequent addition of Cd^2+^ (20 μM) (blue bar) (b) Variation of absorption values of DMBA (20 μM) and DMBA+Cd^2+^ (20 μM) at various pH values.

The detection of the target cation in the presence of other metal ions in real sample is an important assay. Competition experiments were performed to confirm the high selectivity of the detection system. First, the meal ions (200 μM) such as Cu^2+^ , Cr^3+^, Cu^+^, Na^+^, Hg^2+^, Mg^2+^, Ca^2+^, Zn^2+^ , Ag^+^, Pb^2+^, K^+^, Co^2+^, Fe^2+^, Mn^2+^ and Ni^2+^ were preincubated with DMBA (20 μM). As expected, no remarkable change was observed (red bars in Figure 2a). However, the addition of Cd^2+^(20 μM) to each of them resulted in an increase in the absorption intensity at 488 nm (blue bars in Figure 2a). These results show that none of the metal ions tested affect the sensing properties of DMBA to Cd^2+^. Moreover, the effects of pH on the stability of the sensor and its Cd^2+^ complex were investigated and monitored by absorption spectra in a pH range from 1 to 10. The pH of the solutions was adjusted by adding HCl (0.1 m) and NaOH (0.1M) into the solutions. As depicted in Figure 2b, the sensor, DMBA, is not stable at pH 1-2 and gives response to H+ ions at pH 3. The absorbance intensity of DMBA remains constant at pH between 4 and 9, which indicates that Cd^2+^can be detected with DMBA in the environmental pH 4–9.

**Figure 3:**
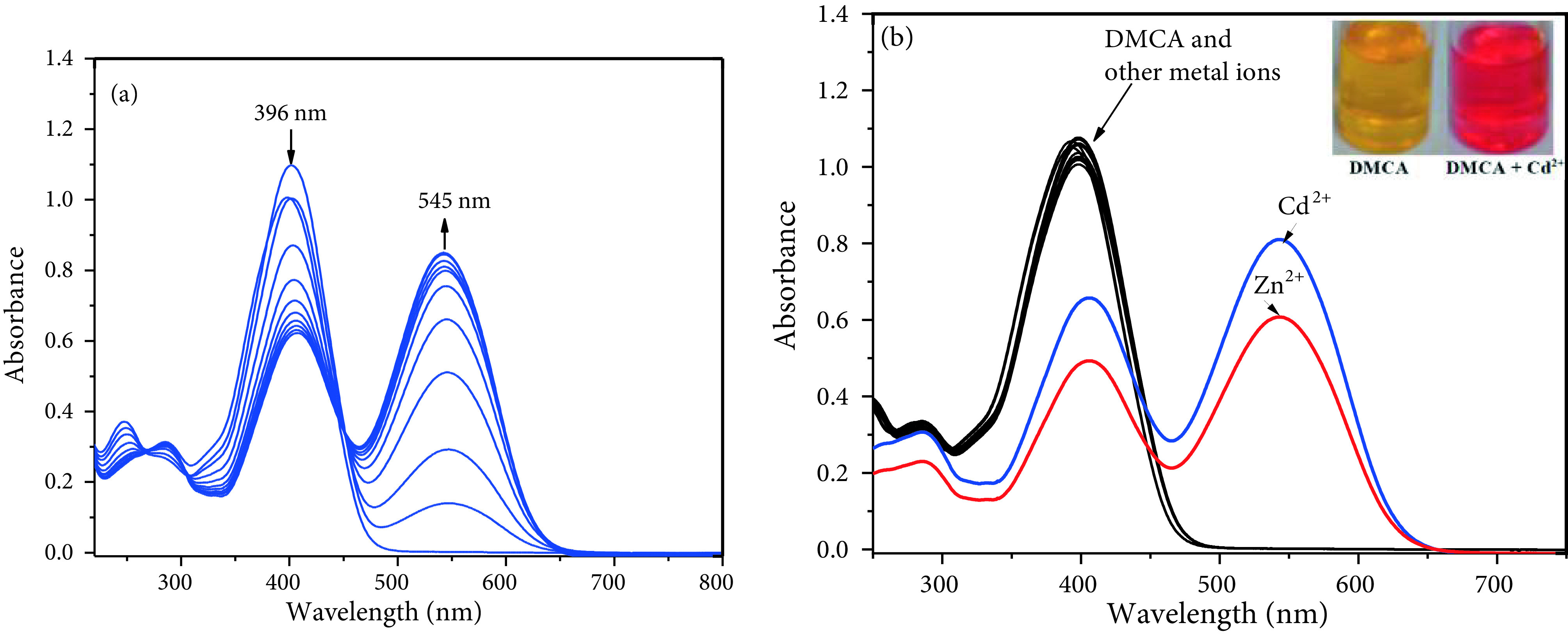
(a) Absorption spectra of 20 μM DMCA with gradual addition of CdCl_2_ (0, 2, 4, 6, 8, 10, 12, 14, 16, 18, 20, 25, 30, 35, and 40 μM, respectively) in ACN/Tris-HCl buffer (10 mM, pH 7.32, v/v 2:1) (b) Absorption spectra of DMCA (20 μM) with various metal ions (20 μM for Cd^2+^, Cu^2+^, Ni^2+^, Mn^2+^, Hg^2+^, Zn^2+^, Ag^+^, Pb^2+^, Fe^3+^, Co^2+^, Fe^2+^, Cu^+^ and Cr^3+^; 50 μM for Ca^2+^, Mg^2+^, K^+^, and Na^+^) in ACN/Tris-HCl buffer (10 mM, pH 7.32, v/v 2:1) (inset: colour changes of DMCA with Cd^2+^) .

We repeated the same selectivity experiments for DMCA. The absorption spectral changes of DMCA after coordination with Cd^2+^in ACN/Tris-HCl buffer (10 mM, pH 7.32, v/v 2:1) were studied first. As seen in Figure 3a, the solution of DMCA alone (20 μM) exhibits an absorption maximum at 396 nm (ε = 5.08 ×104 M^-1^ cm^-1^, only DMCA); as predicted, the maximum absorption wavelength of DMCA (396 nm) is longer than that of DMBA (349 nm) because of the 1 extra double bond. With addition of Cd^2+^to DMCA, the absorbance at 396 nm decreased, while the absorbance at 545 nm (ε = 4.05 ×104 M^-1^ cm^-1^, DMCA and Cd^2+^1:1 ratio) increased accordingly (>64-fold with 1.0 equivalent of Cd2+) (Figure 3a). This apparent bathochromic shift can be explained with the coordination of Cd^2+^. Furthermore, an isosbestic point around 449 nm showed that free sensor molecules convert to Cd^2+^-complex. Moreover, this bathochromic shift of 149 nm resulted in a colour change from yellow to reddish (Figure 3b inset). In contrast, other metal ions such as Cu^2+^ , Cr^3+^, Cu^+^, Na^+^, Hg^2+^, Mg^2+^, Ca^2+^, Ag^+^, Pb^2+^, K^+^, Co^2+^, Fe^2+^, Mn^2+^ and Ni^2+^ did not give response to DMCA (20 μM) (Figure 3b). The sensor also responded to Zn^2+^ with weaker absorption intensity (~48-fold with 1.0 equivalent of Zn^2+^) .

**Figure 4:**
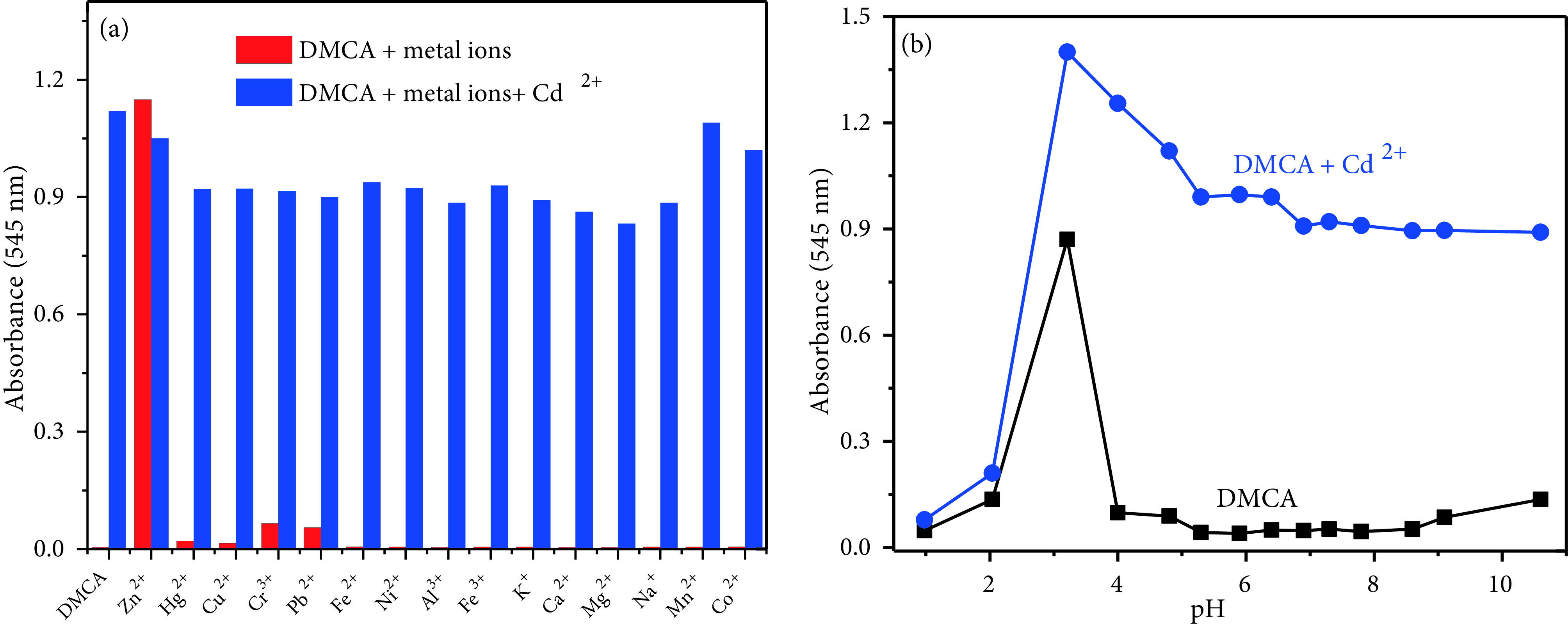
(a) Absorbance values of DMCA (20 μM) with various metal ions (200 μM) (red bar) and the subsequent addition of Cd^2+^ (20 μM) (blue bar) (b) Variation of absorption values of DMCA (20 μM) and DMCA+Cd^2+^ (20 μM) at various pH values.

The competing selectivity of DMCA as a colorimetric sensor for Cd^2+^sensing was controlled with various metal cations. First, the meal ions (200 μM) such as Cu^2+^ , Cr^3+^, Cu^+^, Na^+^, Hg^2+^, Mg^2+^, Ca^2+^, Zn^2+^ , Ag^+^, Pb^2+^, K^+^, Co^2+^, Fe^2+^, Mn^2+^ , and Ni^2+^ were preincubated with DMCA (20 μM). As expected, no remarkable change was observed (red bars in Figure 4a). The naked-eye detection of Cd^2+^by DMCA was not interfered by other metal ions tested, while Zn^2+^ increased the absorption intensity at 545 nm (blue bars in Figure 4a). The stability of the sensor at different pHs (1–10) was also investigated and monitored by UV-vis spectroscopy. The pH of the solutions was adjusted by adding HCl (0.1 M) and NaOH (0.1 M) into the solutions. The absorption intensities of DMCA and DMCA + Cd^2+^at different pH values were plotted in Figure 4b. Cd^2+^ion can be detected with DMCA in the environmental pH range of 4–10.

### 3.3. Investigation of complexation between the sensors and Cd2+

**Figure 5:**
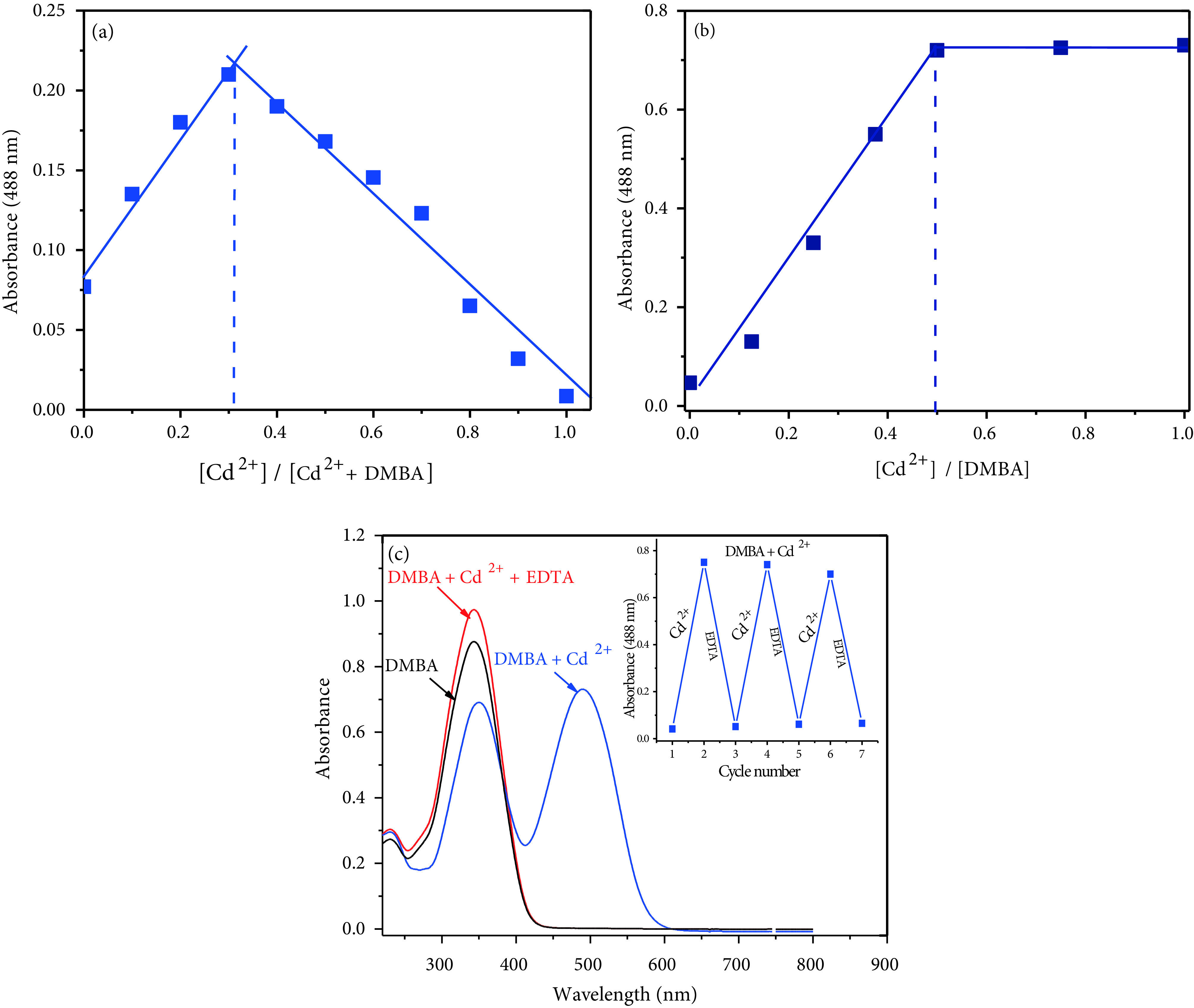
(a) Job’s plot (b) Titration of 20 μM DMBA with gradual addition of CdCl_2_ (0, 2, 4, 6, 8, 10, 12, 14, 16, 18, 20, 25, 30, 35, and 40 μM, respectively) in ACN/Tris-HCl buffer (10 mM, pH 7.32, v/v 2:1) (c) UV-vis spectra showing reversibility of DMBA to Cd^2+^ ions by EDTA.

**Scheme 2:**
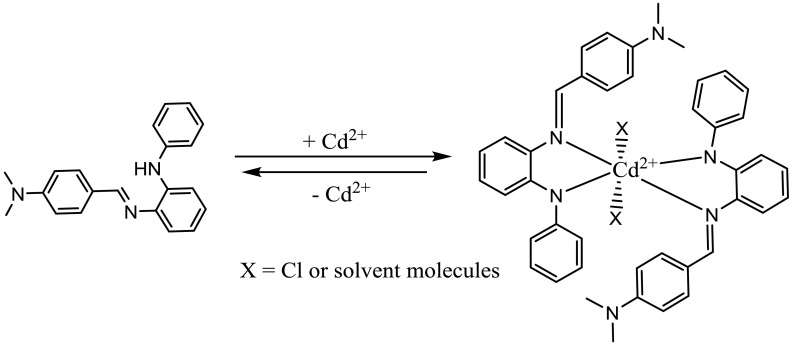
Proposed reversible binding mechanism between DMBA and Cd^2+^.

In order to confirm the binding stoichiometry between the sensors, DMBA and DMCA, and Cd^2+^, Job’s method and UV-vis titration values were used. As shown in Figure 5a (Job’s plot), DMBA/Cd^2+^molar fractions represented a maximum absorption peak (at 488 nm) when it was close to 0.33, which indicates that the binding between DMBA and Cd^2+^was in 2:1 stoichiometry. A titration curve (a plot of DMBA versus Cd^2+^concentration) was also used to determine the binding stoichiometry between DMBA and Cd^2+^. As seen in the inset in Figure 5b, the DMBA/Cd^2+^molar ratio (at 488) reached a plateau when 0.5 equivalent of Cd^2+^was added, suggesting the formation of a 2:1 DMBA-Cd^2+^complex. The binding constant between Cd^2+^and DMBA was determined by a previously reported method [32], with absorption values at 488 nm, and was calculated to be 2.65 ×10^12^ M^-2^ . Furthermore, the reversibility of the binding between the sensor and Cd^2+^was examined. A solution of EDTA (1.0 equivalent) was added to the complex solution of DMBA and Cd^2+^. The absorption signal at 488 nm disappeared and the peak at 349 nm increased (Figure 5c). The absorbance changes were almost reversible even after several cycles with the equivalent addition of Cd^2+^and EDTA (Figure 5c inset). These results indicated that the binding between DMBA and Cd^2+^is reversible. Scheme 2 shows the possible structures for this process.

**Figure 6:**
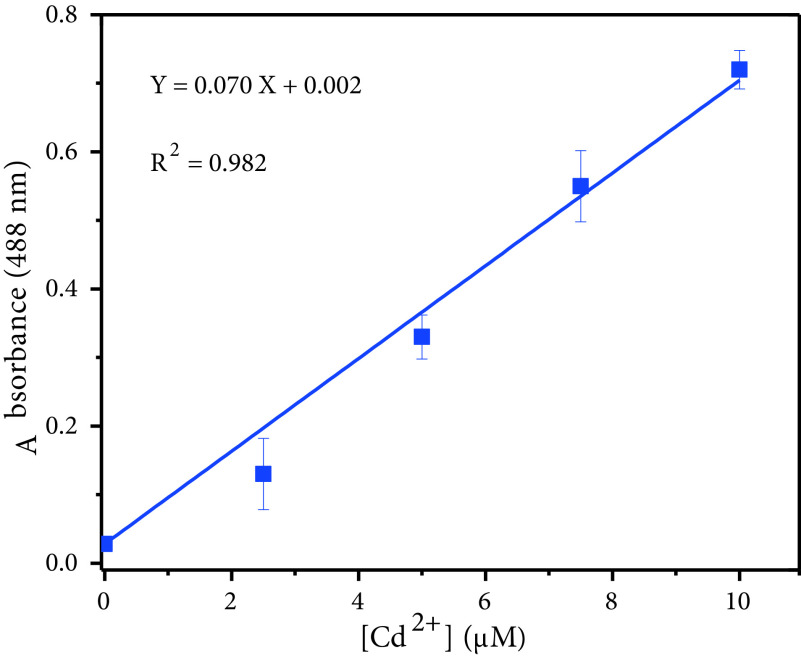
Linear relationship between absorbance intensity and Cd^2+^ concentration (0–20 μM).

Moreover, the linear concentration range and the detection limit of DMBA were obtained. The absorption intensity (at 488 nm) was linearly dependent on the concentration of Cd^2+^in the range from 0 to 10 μM (R^2^ = 0.982). The detection limit was calculated to be 0.438 μM based on 3σ/k (Figure 6) via absorption-based measurement.

**Figure 7:**
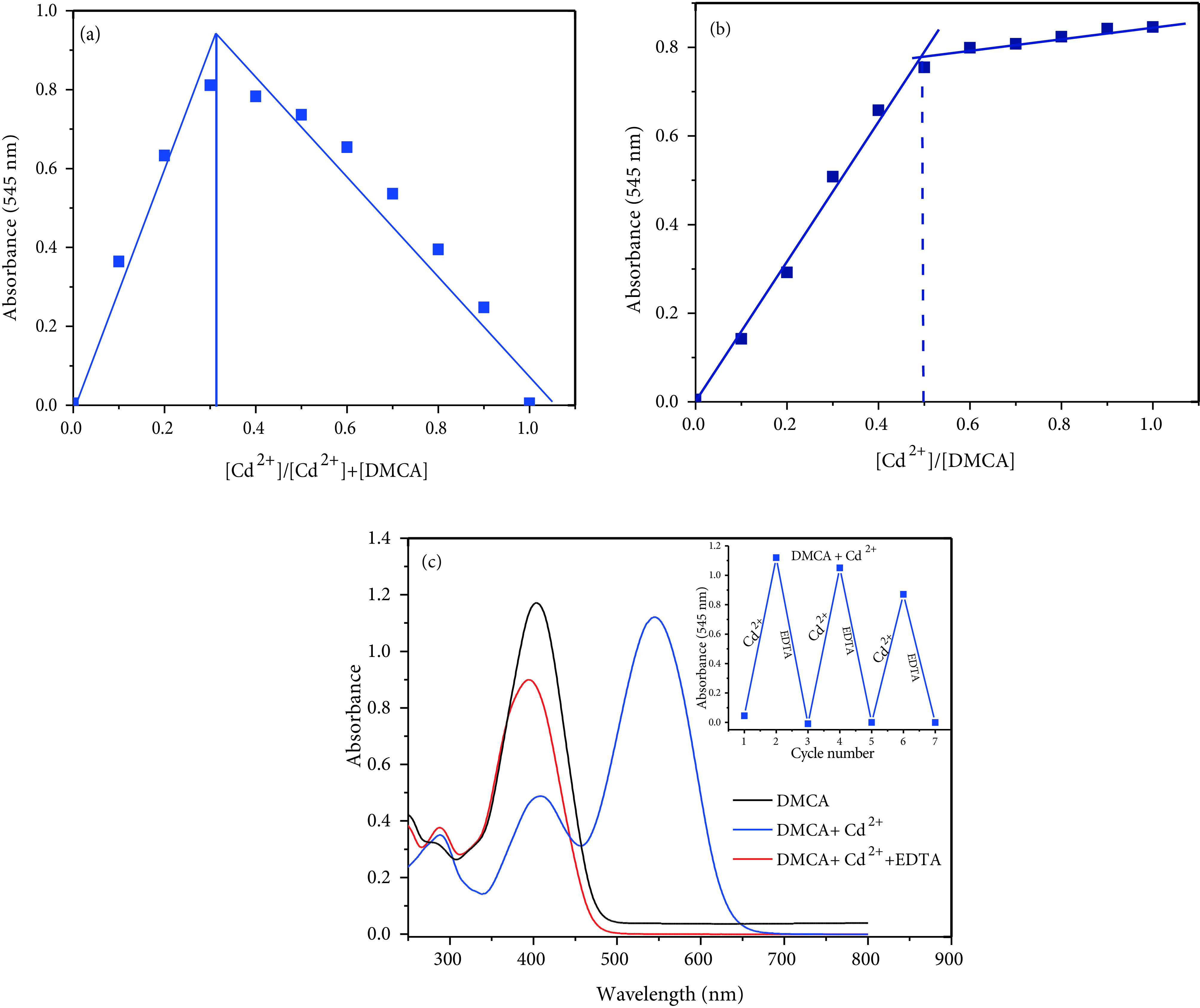
(a) Job’s plot (b) Titration of 20 μM DMCA with gradual addition of CdCl_2_ (0, 2, 4, 6, 8, 10, 12, 14, 16, 18, 20, 25, 30, 35, and 40 μM, respectively) in ACN/Tris-HCl buffer (10 mM, pH 7.32, v/v 2:1) (b) UV-vis spectra showing reversibility of DMCA to Cd^2+^ ions by EDTA.

Job’s method and UV-vis titration were also used to understand binding mode between DMCA and Cd^2+^. As shown in Figure 7a (Job’s plot), DMCA/Cd^2+^molar fractions represented a maximum absorption peak (at 545 nm) when it was close to 0.33, which indicated that the binding between DMCA and Cd^2+^was in 2:1 stoichiometry. As seen in Figure 7b, the absorption band at 545 nm increased gradually up to 0.5 equivalent of Cd^2+^ion respectively, suggesting the formation of a 2:1 DMCA-Cd^2+^complex. The binding constant between Cd^2+^and DMCA was determined with absorption values at 545 nm, and was calculated to be 4.95 ×10^12^ M^-2^ .

**Scheme 3:**
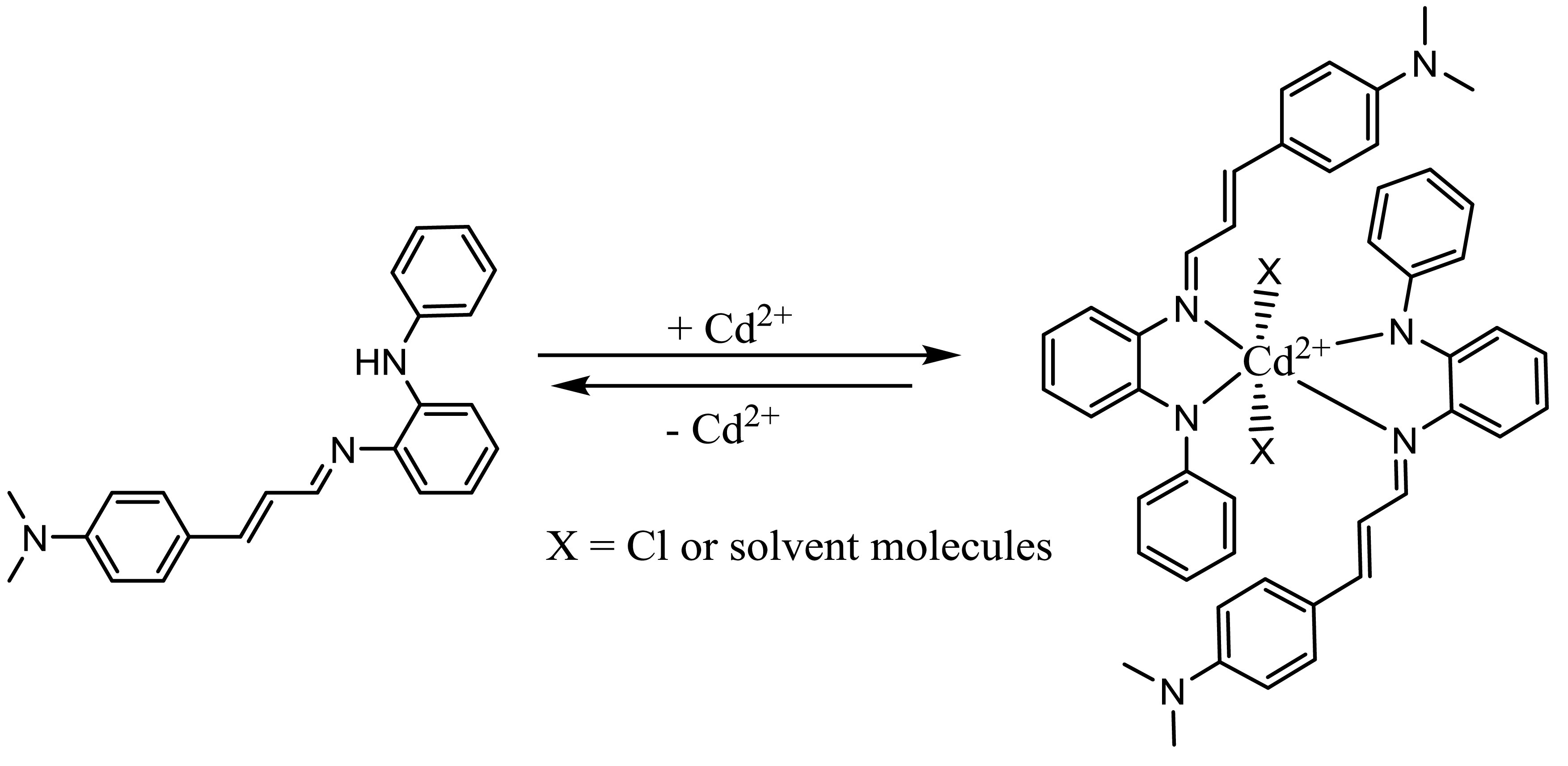
Proposed reversible binding mechanism between DMCA and Cd^2+^.

To ascertain the reversibility of the sensing mechanism of the sensor DMCA, the solution of EDTA (1.0 equivalent) was added to the complex solution of the sensor and Cd^2+^. The absorption signal at 545 nm disappeared and the peak at 396 nm increased (Figure 7b). After addition of Cd^2+^again to the mixture containing DMCA, Cd^2+^, and EDTA, the previous intensity of absorption was almost recovered. Meanwhile, the red solution immediately turned yellow. These cycles were repeated 3 times with the consecutively addition of Cd^2+^/EDTA (Figure 7c inset). These results indicated that the binding between DMCA and Cd^2+^is reversible. Scheme 3 shows the possible structures for this process.

**Figure 8:**
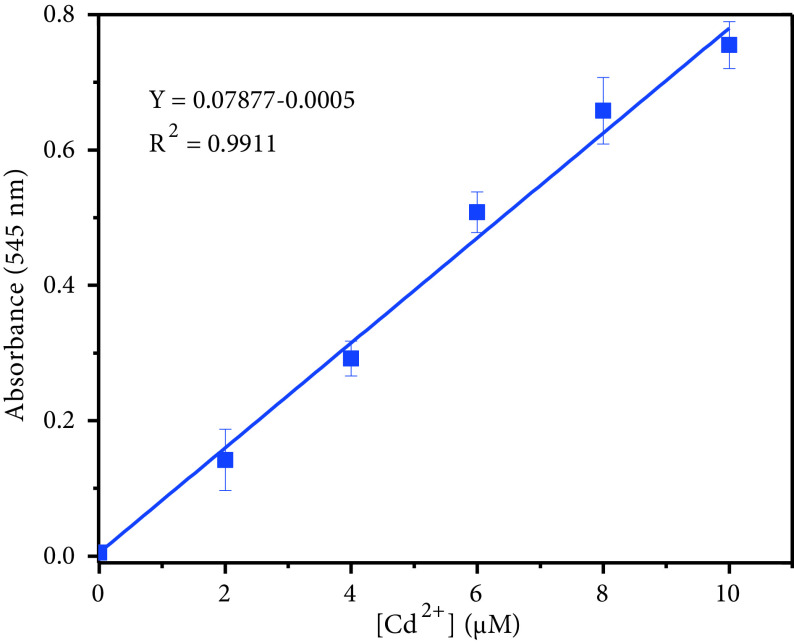
Linear relationship between absorbance intensity and Cd^2+^2+ concentration (0–10 μM).

The linear concentration range and the detection limit of DMCA were also studied. The absorption intensity (at 545 nm) was linearly dependent on the concentration of Cd^2+^in the range from 0 to 10 μM (R^2^ = 0.991). The detection limit was calculated to be 0.102 μM based on 3σ/k (Figure 8) via absorptionbased measurement.

To evaluate the analytical applicability of the sensors, DMBA and DMCA, they were applied for determination of Cd^2+^ions in tap water samples. With the calibration plots of the sensors (Figure 6 and Figure 8), each sample was analysed 3 times and the recovery values were calculated (Table 1). These results showed the suitability and applicability of the sensors for the detection of Cd^2+^in real samples.

**Table 1:** Measurement of Cd^2+^ in tap water samples.

Sensor	Sample	Cd^2+^ Added (mg/L)	Cd^2+^ found (mg/L)	Recovery (%)	RSD (n = 3) (%)
DMBA	Tap water	0	0	-	-
	2	2.05 ± 0.28	102.5	0.37
	5	4.42 ± 0.38	88.4	0.52
	10	9.62 ± 0.14	96.2	2.57
DMCA	Tap water	0	0	-	-
		2	2.02 ± 0.28	101	1.03
		5	5.25 ± 0.31	105	1.88
		10	9.92 ± 0.55	99.2	2.73


Condition: [sensors, (DMBA and DMCA)] = 20 μM in ACN/ Tris-HCl buffer (10 mM, pH 7.32, v/v 2:1)

In recent years, several colorimetric/fluorescent sensors have been developed for the detection of Cd^2+^ions. Compared to some selected sensors, the sensors, DBMA and DMCA, exhibit an excellent ability to detect Cd^2+^ions by changes in both colour and UV-vis absorption spectra with a low detection limit in the presence of various metal ions in aqueous media, as presented in Table 2.

**Table 2:** Comparison of several colorimetric sensors for the detection of Cd^2+^.

Ref.	Testing media	Response time	Response	İnterferences	Reproducibility	Binding constant	Detection limit
[24]	EtOH/H_2_O (1:1, v/v)	NA	Colorimetric/ Fluorescence	Zn^2+^, Pb^2+^	Reversible	1.17 x 10^5^M^-1^	0.073 μ M
[33]	Buffer-acetonitrile	NA	Colorimetric/ Fluorescence	Zn^2+^, Co^2+^	NA	5.60 x 10^5^M^-1^	0.120 μ M
[34]	DMSO	NA	Colorimetric	F^-^ Hg^2+^	Reversible	4.73 x 10$^{3}$ M^-1^	1.03 μ M
[35]	HEPES-buffered solution (20 mM, CH_3_CN: H_2_O, 3:7, v/v, pH 7.0)	NA	Colorimetric/ Fluorescence	None	Reversible	9.56 x 10^5^M^-1^	0.058 μ M
[36]	EtOH/H_2_O (1:1 v/v)	NA	Colorimetric/ Fluorescence	Zn^2+^	Reversible	NA	1.10 μ M
[37]	ACN/HEPES buffer (10 mM, pH: 7.05, v/v 1:1	< 1 min	Colorimetric/ Fluorescence	Cu^2+^, Co^2+^	Reversible	2.70 x 10^7^ M^-1^	0.218 μ M
[38]	10 μ M HEPES buffer solution (pH 7.54),	NA	Colorimetric/ Fluorescence	None	NA	2.33 x 10^5^M^-1^	0.018 μ M
[39]	CH_2_Cl_2_/CH_3_CN (1:9, v/v).	NA	Colorimetric/ Fluorescence	None	Reversible	10$^{3}$-10$^{4}$ (1:3 complex)	NA
[40]	DMF/H_2_O (9:1, v/v)	< 1 min	Colorimetric/ Fluorescence	Zn^2+^, Ni^2+^, Mn^2+^	Reversible	4.98 x 10$^{4\, }$M^-1^	0.861 μ M
DMBA This study	ACN/Tris-HCl buffer (10 mM, pH 7.32, v/v 2:1)	< 1 min	Colorimetric	None	Reversible	2.65 x 10^12^ M^-2^	0.438 μ M
DMCA This study	ACN/Tris-HCl buffer (10 mM, pH 7.32, v/v 2:1)	< 1 min	Colorimetric	Zn^2+^	Reversible	4.95 x 10^12^ M^-2^	0.102 μ M

PEC = NA: not available

## 4. Conclusion

We presented 2 new Schiff base derivatives, a benzaldehyde-based sensor (DMBA) and a cinnamaldehydebased sensor (DMCA), for colorimetric sensing of Cd^2+^ions in aqueous solutions. The sensors depicted visible absorption characteristics that range from colourless to orange for DMBA and yellow to reddish for DMCA. The sensors had a 2-nitrogen Cd^2+^-receptor moiety and coordinates with Cd^2+^in a 2:1 binding mode with a reversible response. The binding constant of the complexes was calculated as 2.65 ×10^12^ M^-2^ for DMBA and 4.95 ×10^12^ M^-2^ for DMCA. The detection limits of DMBA and DMCA were calculated via absorption-based measurement and found to be 4.38 ×10^-7^M and 1.02 ×10^-7^, respectively, which gave a marked sensitivity towards Cd^2+^. For the practical application, the sensors were applied to real samples for identifying Cd^2+^in tap water. Therefore, the sensors, DMBA and DMCA, could serve as a colorimetric sensor for the detection of Cd^2+^in aqueous solutions.
